# Editorial: Type I Chaperonins: Mechanism and Beyond

**DOI:** 10.3389/fmolb.2018.00072

**Published:** 2018-07-31

**Authors:** Adina Breiman, Abdussalam Azem

**Affiliations:** ^1^School of Plant Sciences and Food Security, Tel Aviv, Israel; ^2^School of Neurobiology, Biochemistry and Biophysics, George S. Wise Faculty of Life Sciences, Tel Aviv University, Tel Aviv, Israel

**Keywords:** chaperonin 60, chaperonins, GroEL, HSP10, GroES

Chaperone proteins control almost all aspects of proteostasis, such as protein synthesis, translocation, folding, and degradation. As such, chaperones accompany every protein from its birth until its death. Chaperonins constitute a highly conserved subgroup of molecular chaperones that is divided into two groups, Type I and Type II. For Type I chaperonins, the protein folding function is mediated by the Hsp60 (also known as Cpn60) chaperonin, which serves as a folding chamber for denatured protein, assisted by its 10 kDa co-chaperonin, Hsp10 (or Cpn10). For Type II chaperonins, the protein folding function is handleed by a single Hsp60 protein, CCT/TRiC (Horwich et al., 2006, p. 5464; Dekker et al., 2011; Skjaerven et al., 2015). Several important milestones are worth mentioning that led to our current understanding of the molecular of function of Type I chaperonins. The latter were discovered in the 1970s as bacterial host proteins that are essential for the assembly of phage particles (Georgopoulos et al., 1973). During the same period, the heat shock response of some chaperones was discovered (Ritossa, 1962). Conditions known to compromise protein folding. Additional *in vivo* studies showed that chaperonins are key players in the assembly process of RuBisCO in plants and that they are important for the folding of newly translocated proteins into the mitochondrial matrix as well (Hemmingsen et al., 1988; Roy et al., 1988; Cheng et al., 1989; Goloubinoff et al., 1989).

These discoveries led to general recognition of Type I chaperonins as important protein nano machines that play a key role in cellular protein folding and assembly. *In vitro* reconstitution of their protein folding activity using denatured dimeric RuBisCO as a model system opened the door to a new field of research, which focused on *in vitro* mechanistic aspects of chaperonin function (Goloubinoff et al., 1989). The friendly nature of the Escherichia coli chaperonins, in particular the profound stability of the protein oligomers, enabled their extensive investigation, which established them as the prototype chaperonin model system. Notably, the preponderance of research in the field focused on mechanistic aspects of this bacterial chaperone system.

With time, investigation of chaperonins from chloroplasts, mitochondria, and numerous additional bacterial strains, revealed a wide range of divergence from the *E. coli* paradigm. The vast diversity among chaperonins and atypical systems such as those as discovered in bacteriophages, is reviewed in two manuscripts (Ansari and Mande; Bhatt et al.).

In the case of chloroplast chaperonins, the most striking observation was that these chaperonins assemble into hetero-oligomeric tetradecamers that are composed of several homologous subunits, in contrast to the homo oligomeric nature of bacterial chaperonins. The chloroplast chaperonins are the subject of three manuscripts in this research topic (Zhao and Liu; Vitlin Gruber and Feiz; Vitlin Gruber et al.). Two of them highlight the sophisticated RuBisCO assembly pathway, with new assembly factors identified in recent years, and the complexity of the chloroplast chaperonin system. Recent discoveries in the field represent an important step toward possibly engineering more efficient RuBisCO thereby potentially increasing crop yield.

With regard to mitochondrial chaperonins, these were also found to exhibit unique structural properties and retain unexpected extra-organelle moonlighting functions. As such, they were found to function in a variety of processes, including signal transduction events that may regulate immunity and inflammation (Athanasas-Platsis et al., 2004; Grundtman et al., 2011; Jia et al., 2011; Juwono and Martinus, 2016). Mitochondrial Hsp60 was suggested to adopt variations in its oligomeric state, in a nucleotide and concentration-dependent manner that may affect its function. Vilasi et al. review the oligomeric variability of mitochondrial Hsp60 and its link to functions that are not related to protein folding (cytosolic and extracellular) (Vilasi et al.). Due to their extra mitochondrial functions, in particular in tumors, Hsp60 has been considered to be a potential target for anticancer drugs. Meng et al. provide an updated review of available compounds that inhibit or affect the function of Hsp60 chaperonins (Meng et al.), with an eye toward using them as anticancer drugs.

In the biotechnology arena, O'Neil et al. developed a highly sophisticated system that utilizes immobilized GroEL on sensors for the detection of aggregated proteins among the various species in solution (O'Neil et al.).

For almost three decades, research on the bacterial GroEL/GroES chaperonin molecular mechanism of function has been central in the field of chaperone proteins (Thirumalai and Lorimer, 2001; Horwich et al., 2006; Gruber and Horovitz, 2016; Hayer-Hartl et al., 2016). The identity of active forms during the reaction cycle, whether the symmetrical (GroEL)_14_ ((GroES)_7_)_2_ (also named footballs) or the asymmetrical (GroEL)_14_(GroES)_7_ complexes (bullets), the role of chaperonins in the cycle (e.g., passive or active) and the role of ATP (Goloubinoff et al., 2018) all are discussed in several of the contributions, particularly in Weiss et al. The molecular function of the mitochondrial Hsp60/Hsp10 chaperonin system receives special attention in this context. Initially, it was suggested that Hsp60 operates as a single ring (Nielsen and Cowan, 1998; Nielsen et al., 1999), rather than a double ring as suggested for GroEL. In Weiss et al, based on results obtained in several studies, an alternative model was endorsed for the Hsp60 reaction cycle (Weiss et al.). This model proposes that mitochondrial Hsp60 alternates between single ring and double ring structures. This “equatorial split” is probably essential for the proper function of the mitochondrial system. Notably, such equatorial split mechanism was originally suggested for Thermus Thermophilus (Ishii et al., 1995), proposed also for GroEL (Taguchi, 2015) and recently received additional experimental support (Yan et al., 2018). Notably, preventing the equatorial split of the rings, by either formation of S-S bonds (Yan et al., 2018) or covalent fusion, still allows for significant protein folding activity by GroEL (Farr et al., 2003). Thus, the functional significance of the ring split for GroEL still requires further investigation.

## Author contributions

All authors listed have made a substantial, direct and intellectual contribution to the work, and approved it for publication.

### Conflict of interest statement

The authors declare that the research was conducted in the absence of any commercial or financial relationships that could be construed as a potential conflict of interest.
